# Stress-Associated Phenylpropanoid Metabolism and Nutritional Composition in Wild vs. MeJA-Elicited In Vitro *Hypericum perforatum* and *Portulaca oleracea*

**DOI:** 10.3390/metabo16030161

**Published:** 2026-02-28

**Authors:** Gulmira Zhakupova, Assem Sagandyk, Tamara Tultabayeva, Aknur Muldasheva, Kadyrzhan Makangali, Aigerim Akhmetzhanova

**Affiliations:** Department of Food Technology and Processing Products, S. Seifullin Kazakh Agrotechnical Research University, Astana 010000, Kazakhstan

**Keywords:** phenylpropanoid pathway, secondary metabolites, methyl jasmonate, elicitation, stress response, *Hypericum perforatum*, *Portulaca oleracea*, in vitro cultivation

## Abstract

Background/Objectives: The phenylpropanoid pathway in plants plays a pivotal role in the biosynthesis of secondary metabolites in plants, responding to environmental stresses to enhance protective compounds such as phenolic acids and flavonoids. This study compares the phenolic profiles, vitamins, sugars, and mineral elements of *Hypericum perforatum* and *Portulaca oleracea* grown under two contrasting conditions: wild habitats and in vitro cultures on Murashige–Skoog medium supplemented with methyl jasmonate (MeJA, 25–50 µM). Methods: Aerial parts were extracted with 70% ethanol and analyzed for phenolic profiles (rutin, caffeic acid, chlorogenic acid, quercetin), proximate composition, free sugars, vitamins, and mineral elements (*n* = 3, ANOVA/Tukey, *p* < 0.05). In vitro cultures were maintained under MeJA-elicited conditions; however, the present design does not allow for the separation of MeJA-specific effects from general in vitro growth conditions. Results: Wild samples showed higher phenolic contents (e.g., rutin in *Hypericum perforatum*: 22.224 ± 0.65 mg/g vs. 15.190 ± 0.311 mg/g in vitro; quercetin in *Portulaca oleracea*: 0.874 ± 0.157 mg/g vs. 0.444 ± 0.157 mg/g), highlighting the stress-induced activation of secondary metabolism in natural environments. Conclusions: Overall, the data indicate that wild-growing plants accumulate higher levels of key phenylpropanoids than MeJA-elicited in vitro cultures, underscoring the complexity of reproducing natural stress-associated metabolic patterns under controlled conditions.

## 1. Introduction

The phenylpropanoid pathway is a central metabolic route in plants responsible for synthesizing a wide array of secondary metabolites, including phenolic acids (e.g., caffeic and chlorogenic acids) and flavonoids (e.g., rutin and quercetin) [1,2]. Initiated by the deamination of phenylalanine via phenylalanine ammonia-lyase (PAL), the pathway proceeds through cinnamate 4-hydroxylase (C4H) and 4-coumarate:CoA ligase (4CL) to form p-coumaroyl-CoA, a precursor branching into flavonoids via chalcone synthase (CHS) and phenolic acids via additional hydroxylations [2]. These compounds play crucial roles in plant adaptation to environmental stresses, such as abiotic factors (salinity, drought, UV radiation) and biotic interactions (pathogens), by acting as antioxidants, signaling molecules, and structural reinforcements [1,3].

Stress significantly upregulates the pathway: for instance, osmotic stress (e.g., NaCl) in Salicornia species increases rutin and quercetin levels by 2–3-fold. In wild-growing plants, natural stressors inherently activate this response, leading to higher metabolite accumulation compared to stress-free conditions [4]. In vitro cultivation, such as on Murashige–Skoog (MS) medium, provides a platform for metabolic engineering but often results in lower levels of secondary metabolites due to the absence of environmental cues [5].

*Hypericum perforatum* (St. John’s wort) and *Portulaca oleracea* (purslane) are model species for studying phenylpropanoids: *H. perforatum* is rich in hypericins, rutin, and quercetin, contributing to its stress resilience [6,7,8], while *P. oleracea* accumulates chlorogenic acid and flavonols, aiding in drought tolerance [9,10,11,12]. Elicitors like methyl jasmonate (MeJA) mimic stress by activating jasmonate signaling pathways, potentially boosting PAL and CHS expression [13].

Although numerous studies have characterized either phenolic profiles or selected nutritional traits or both combined *H. perforatum* and *P. oleracea*, there is a lack of integrated data sets that jointly compare phenylpropanoids, vitamins, sugars, and mineral elements in wild plants and MeJA-elicited in vitro cultures of these species, particularly in a food-oriented context.

In this work, we compare phenolic profiles (rutin, caffeic acid, chlorogenic acid, quercetin) and nutritional composition (proximate, vitamins, sugars, minerals) between wild-growing plants and in vitro cultures on MeJA-supplemented MS medium. We specifically assess whether controlled, elicited in vitro conditions can reproduce the stress-associated metabolic patterns observed in nature. Therefore, the specific aim of this study was to compare phenolic profiles together with nutritional (proximate, vitamin, sugar, and mineral) composition in wild-growing and MeJA-elicited in vitro cultures of *H. perforatum* and *P. oleracea* and to interpret these differences in the context of phenylpropanoid pathway activity and their potential relevance for food applications.

## 2. Materials and Methods

### 2.1. Plant Material and Cultivation Conditions

**Figure 1:**
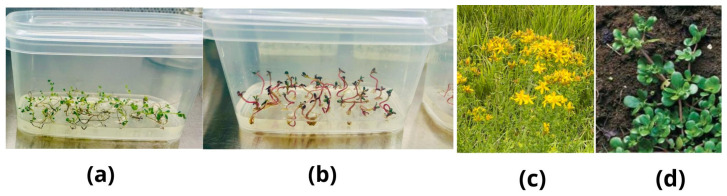
Samples of plants used in this study: (**a**)—in vitro *H. perforatum*; (**b**)—in vitro *P. oleracea*; (**c**)—wild *H. perforatum*; (**d**)—wild *P. oleracea*. Figure 1 illustrates the two contrasting growth systems (wild habitat vs. MeJA-elicited in vitro culture) from which plant material was collected for subsequent compositional analyses.

Seeds of *H. perforatum* (St. John’s wort) and *P. oleracea* (purslane), illustrated in Figure 1, were obtained from the Institute of Engineering and Food Technology, S. Seifullin Kazakh Agrotechnical Research University (Astana, Kazakhstan). For the in vitro experiment, plant explants were cultivated on Murashige and Skoog (MS) basal medium (pH 5.8) containing 3% *w*/*v* sucrose and 0.8% agar. The culture medium was autoclaved at 121 °C for 20 min, cooled to approximately 50 °C, and then supplemented under sterile conditions with filter-sterilized methyl jasmonate (MeJA) stock solution to obtain final concentrations of 25 or 50 µM before pouring it into sterile Petri dishes. Cultures were maintained in a growth chamber at 24 ± 2 °C with a photoperiod of 16 h light/8 h dark and a light intensity of 50 µmol m^−2^ s^−1^. Control plants were grown wild outside of the city and harvested in the middle of summer 2025. After 30 days of cultivation, aerial parts of MS-grown plants were harvested. All collected samples were washed, lyophilized, and ground into fine powder using a laboratory mill IKA A11 basic (IKA Werke GmbH & Co. KG, Staufen, Germany). All in vitro explants were cultivated on MeJA-supplemented MS medium for 30 days. Non-elicited in vitro controls were not included to allow for focused comparison between wild plants and MeJA-elicited cultures.

### 2.2. Preparation of Plant Extracts

**Figure 2:**
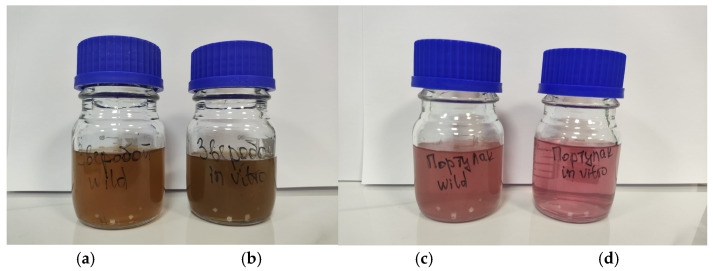
Extracts of plants: (**a**)—extract of wild *H. perforatum*; (**b**)—extract of in vitro *H. perforatum*; (**c**)—extract of wild *P. oleracea*; (**d**)—extract of in vitro *P. oleracea*.

Dried 5 g powders were extracted with 50 mL of 70% ethanol *v*/*v* at 45 °C for 2 h under constant stirring at 300 rpm. Extracts were filtered through Whatman No. 1 paper and concentrated under reduced pressure (Büchi Rotavapor R-300, Flawil, Switzerland) at 40 °C, followed by drying in a vacuum oven to constant weight. Extracts were stored in amber vials at 4 °C until analysis (Figure 2).

### 2.3. Determination of Proximate Composition

The contents of moisture, ash, crude protein, fat, and carbohydrates were determined according to AOAC methods. Protein was measured by the Kjeldahl method at N × 6.25, fat by Soxhlet extraction, ash by muffle furnace at 550 °C, and carbohydrates by the difference. All measurements were expressed on a dry matter basis.

### 2.4. Determination of Polyphenols and Flavonoids

Total polyphenol content (TPC) was determined by the Folin–Cocalteu method and expressed as mg gallic acid equivalents (GAE)/100 g dry weight (DW). Total flavonoid content (TFC) was determined using the aluminum chloride colorimetric method and expressed as mg quercetin equivalents (QE)/100 g DW. UV–Vis spectrophotometry was performed on a Shimadzu UV-1900 spectrophotometer (Kyoto, Japan).

### 2.5. UHPLC-QToF-MS Analysis of Phenolic Compounds

Phenolic profiles were analyzed using a UHPLC-QToF-MS system (Agilent 1290 Infinity II UHPLC coupled with Agilent 6546 QToF-MS, Santa Clara, CA, USA). Separation was carried out on a C18 column 2.1 × 100 mm, 1.7 µm with a mobile phase of A 0.1% formic acid in water and B 0.1% formic acid in acetonitrile under gradient conditions 5–95% B over 30 min. The flow rate was 0.3 mL/min, injection volume 5 µL, and detection wavelength 280 nm. The identification and quantification of phenolics were achieved using external standards.

### 2.6. Determination of Free Carbohydrates

Free sugars were analyzed using high-performance liquid chromatography (HPLC) with refractive index detection (Agilent 1260 Infinity, Santa Clara, CA, USA). Separation was achieved on a Rezex RCM-Monosaccharide Ca^2+^ column 300 × 7.8 mm at 80 °C with distilled water as the mobile phase at 0.6 mL/min. Quantification was performed using calibration curves for analytical standards of glucose, fructose, and sucrose.

### 2.7. Determination of Vitamin Content

Vitamins B_1_, B_2_, B_3_, B_5_, B_6_, B_9_, and C were determined using HPLC with UV-Vis detection according to AOAC. The analysis was performed on a C18 column 250 × 4.6 mm, 5 µm at 30 °C, using gradient elution with phosphate buffer of pH 6.0 and methanol. Detection wavelengths were 254 nm for B-vitamins and 265 nm for vitamin C. Quantification was based on calibration with certified reference standards (Sigma-Aldrich, St. Louis, MO, USA).

### 2.8. Determination of Macro- and Microelements

The mineral composition was determined using inductively coupled plasma optical emission spectrometry (ICP-OES; Agilent 5110, Santa Clara, CA, USA) after the microwave digestion of 0.5 g of dried extract in 10 mL of HNO_3_ 65%, suprapure. Quality control was verified using certified reference material (NIST 1573a Tomato Leaves). Toxic elements were analyzed by atomic absorption spectrometry (AAS, Shimadzu AA-7000, Kyoto, Japan). Radionuclides ^40^K and ^137^Cs were measured using gamma spectrometry (Canberra HPGe detector, Meriden, CT, USA).

### 2.9. Statistical Analysis

All experiments were conducted in triplicate (*n* = 3). Data are presented as the mean ± standard deviation (SD). A one-way analysis of variance (ANOVA) followed by Tukey’s HSD test was performed using GraphPad Prism 10.0 (GraphPad Software, San Diego, CA, USA). Differences were considered statistically significant at *p* < 0.05.

## 3. Results

### 3.1. Physicochemical Composition of Herbal Extracts

The physicochemical composition of extracts from *H. perforatum* and *P. oleracea* is presented in Table 1. For *H. perforatum*, protein content was 45.46 ± 0.11% in wild samples and 49.20 ± 0.10% in in vitro samples; fat was 16.75 ± 0.07% and 18.10 ± 0.06%, respectively; carbohydrates were 31.67 ± 0.25% and 32.65 ± 0.24%; ash was 6.12 ± 0.05% and 7.05 ± 0.05%. For *P. oleracea*, protein was 34.35 ± 0.12% in wild and 38.10 ± 0.11% in in vitro; fat 8.29 ± 0.02% and 8.90 ± 0.03%; carbohydrates 31.61 ± 0.32% and 34.80 ± 0.28%; ash 25.75 ± 0.05% and 28.20 ± 0.07%.

### 3.2. Total Polyphenols and Flavonoids

Total polyphenol and flavonoid contents are summarized in Table 2. For *H. perforatum*, TPC was 2.70 ± 0.06% in wild and 3.58 ± 0.07% in in vitro; TFC 2.35 ± 0.05% and 3.12 ± 0.06%. For *P. oleracea*, TPC 1.25 ± 0.02% and 1.78 ± 0.03%; TFC 0.98 ± 0.02% and 1.35 ± 0.03%.

### 3.3. Phenolic and Flavonoid Profiles Determined by UHPLC-QToF-MS

The phenolic profiles are shown in Table 3. In wild *H. perforatum* extracts, rutin was 22.224 ± 0.65 mg/g, caffeic acid 31.23 ± 1.66 mg/kg, chlorogenic acid 10.79 ± 1.11 mg/kg, and quercetin 1.773 ± 0.251 mg/g. In in vitro, rutin was 15.190 ± 0.311 mg/g, caffeic acid 29.11 ± 1.48 mg/kg, chlorogenic acid 7.44 ± 0.81 mg/kg, and quercetin 1.196 ± 0.166 mg/g. For wild *P. oleracea*, rutin was 35.440 ± 4.120 mg/g, caffeic acid 12.6 ± 0.2 mg/kg, chlorogenic acid 23.76 ± 1.89 mg/kg, and quercetin 0.874 ± 0.157 mg/g. In in vitro, rutin was 20.445 ± 2.454 mg/g, caffeic acid < 1.0 mg/kg, chlorogenic acid 13.79 ± 1.55 mg/kg, and quercetin 0.444 ± 0.157 mg/g.

### 3.4. Free Carbohydrate Composition

Free carbohydrate contents are presented in Table 4. In wild *H. perforatum*, sucrose was 0.45 ± 0.02 g/100 g, glucose 1.60 ± 0.06 g/100 g, and fructose 2.43 ± 0.07 g/100 g. In in vitro, sucrose was 0.52 ± 0.03 g/100 g, glucose 1.98 ± 0.06 g/100 g, and fructose 3.05 ± 0.08 g/100 g. For wild *P. oleracea*, sucrose was nd, glucose 2.28 ± 0.07 g/100 g, and fructose nd. In in vitro, sucrose was 0.18 ± 0.02 g/100 g, glucose 2.75 ± 0.08 g/100 g, and fructose nd.

### 3.5. Vitamin Composition of Extracts

Vitamin contents are shown in Table 5. In wild *H. perforatum*, B1 was 3.781 ± 0.756 mg/100 g, B2 2.940 ± 1.234 mg/100 g, B3 11.703 ± 2.340 mg/100 g, B5 6.278 ± 1.256 mg/100 g, B6 1.135 ± 0.227 mg/100 g, B9 0.410 ± 0.082 mg/100 g, and C 256.14 ± 87.08 mg/100 g. In in vitro, B1 was 4.920 ± 0.984 mg/100 g, B2 3.860 ± 1.617 mg/100 g, B3 14.850 ± 2.970 mg/100 g, B5 8.200 ± 1.640 mg/100 g, B6 1.490 ± 0.298 mg/100 g, B9 0.540 ± 0.108 mg/100 g, and C 326.00 ± 111.0 mg/100 g. Similar patterns were observed for *P. oleracea*.

### 3.6. Macro- and Microelement Profiles

Mineral compositions are presented in Table 6. In wild *H. perforatum*, Fe was 1.240 ± 0.004 mg/100 g, Zn 6.83 ± 0.31 mg/100 g, Mg 2.30 ± 0.02 mg/100 g, K 181.6 ± 0.8 mg/100 g, Ca 89.73 ± 1.07 mg/100 g, and P 222.13 ± 0.09 mg/100 g. In in vitro, Fe was 0.970 ± 0.003 mg/100 g, Zn 8.21 ± 0.33 mg/100 g, Mg 2.96 ± 0.03 mg/100 g, K 214.7 ± 0.9 mg/100 g, Ca 102.80 ± 1.14 mg/100 g, and P 253.45 ± 0.10 mg/100 g. For wild *P. oleracea*, Fe was 20.50 ± 0.05 mg/100 g, Zn 4.50 ± 0.10 mg/100 g, Mg 750.0 ± 1.5 mg/100 g, K 5500.0 ± 5.0 mg/100 g, Ca 700.0 ± 2.0 mg/100 g, and P 500.0 ± 1.0 mg/100 g. In in vitro, Fe was 27.12 ± 0.06 mg/100 g, Zn 5.50 ± 0.12 mg/100 g, Mg 930.2 ± 0.8 mg/100 g, K 6810.44 ± 2.45 mg/100 g, Ca 884.75 ± 1.50 mg/100 g, and P 612.40 ± 1.20 mg/100 g.

## 4. Discussion

The results demonstrate that wild-growing *H. perforatum* and *P. oleracea* accumulate higher levels of phenolic compounds, vitamins, and certain minerals than their in vitro counterparts elicited with 25–50 μM methyl jasmonate (MeJA) on Murashige–Skoog (MS) medium. Consistent with this pattern, we observed that wild *H. perforatum* and *P. oleracea* contained 46% and 72% higher levels of rutin and chlorogenic acid than their in vitro counterparts (Table 3). Together with the higher TPC/TFC values in wild *H. perforatum* (Table 2), these findings support the view that natural abiotic and biotic stresses induce a stronger activation of phenylpropanoid metabolism than the MeJA-elicited in vitro conditions used here. This disparity underscores the sensitivity of the phenylpropanoid pathway to environmental stresses, where natural cues in wild conditions more effectively activate secondary metabolite biosynthesis than controlled elicitation [1,2].

**Figure 3:**
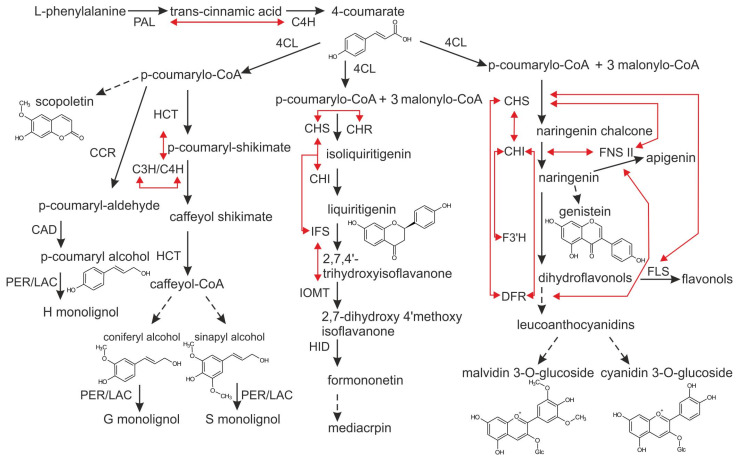
Schematic of phenylpropanoid pathway, illustrating key enzymes (PAL, C4H, 4CL, CHS, CHI, F3H, and FLS for flavonoids; CCR and CAD for lignin) and branches. Black arrows indicate main metabolic steps; red arrows indicate upregulated reactions/enzymes; dashed arrows indicate alternative pathways.

Based on our compositional data (Table 1, Table 2, Table 3, Table 4, Table 5 and Table 6), wild plants accumulated higher levels of several phenylpropanoids and vitamins than MeJA-elicited in vitro cultures. These differences can be interpreted in the context of previously described regulatory mechanisms of the phenylpropanoid pathway. As summarized in Figure 3 and detailed in previous reviews, the phenylpropanoid pathway channels carbon from phenylalanine through PAL, C4H and 4CL into lignin and flavonoid branches, ultimately yielding phenolic acids (e.g., caffeic, chlorogenic) and flavonols (e.g., quercetin, rutin) [1,2,14,15]. We therefore interpret the higher phenolic contents in wild plants as an indication of more strongly activated phenylpropanoid flux under natural stress conditions compared with MeJA-elicited in vitro cultures (Table 3).

Previous work has demonstrated that transcription factors of the MYB–bHLH–WD40 complex, as well as epigenetic and post-translational modifications, can strongly modulate the activity of key enzymes of the phenylpropanoid pathway. Although these mechanisms were not directly assessed in our study, they provide a plausible framework to explain why wild plants, exposed to complex environmental stresses, accumulated higher levels of rutin, quercetin and related phenolics than in vitro cultures (Table 3).

Proximate composition showed modest in vitro increases (e.g., protein 8% higher in *P. oleracea*), as MS nutrients favor primary metabolism, while wild stresses divert resources to phenylpropanoids, reducing growth but increasing ash/mineral content, which serve as cofactors (e.g., Mn for POD in the lignin branch) [16,17,18,19]. Total polyphenols/flavonoids (TPC/TFC) were 32% higher in wild *H. perforatum*, correlating with pathway branching efficiency under stress [20]. Free sugars, elevated in vitro (e.g., glucose 23% higher in *P. oleracea*), act as precursors for glycosylated flavonoids like rutin via UDP-glycosyltransferases, but in wild plants, sugar catabolism supports the energy demand of pathway activation [21]. Vitamins, particularly vitamin C (27% higher in wild *H. perforatum*), contribute to redox homeostasis and may synergize with phenylpropanoids (e.g., ascorbate recycling flavonoids) [22,23,24,25]. Minerals enriched in vitro (e.g., Mg 24% higher in *P. oleracea*) support Mg-dependent enzymes such as CHS, yet wild stresses appear to favor metabolite synthesis over accumulation [25,26].

In wild samples, elevated phenolic levels (e.g., rutin 46% higher in wild vs. in vitro *H. perforatum*; chlorogenic acid 72% higher in wild vs. in vitro *P. oleracea*) are consistent with the stress-induced upregulation of PAL, CHS and FLS reported in other species, which would channel carbon flux toward reactive oxygen species (ROS) scavenging and defense. Natural abiotic stresses (UV, drought, salinity) have been shown to activate transcription factors (MYB/bHLH) and jasmonate/ethylene signaling, thereby enhancing enzyme expression and metabolite diversity in various plants. Although our study did not evaluate these regulatory layers directly, the lower phenolic levels in MeJA-elicited in vitro cultures compared to wild plants suggest that additional or stronger cues may be required to reproduce the wild-type activation of this pathway [13].

Taken together, these data indicate that primary metabolites and minerals provide the resource base and metabolic background for phenylpropanoid biosynthesis and that the different growth conditions modulate how carbon and mineral nutrients are partitioned between primary and secondary metabolism.

Our findings align with Gao et al. (2010) [27], who reported high variability in phenolics (e.g., quercetin, chlorogenic acid) in *H. perforatum* products, with wild samples showing 1.5–2× higher levels than cultivated ones, attributing this to stress-activated PAL/CHS. Similarly, Fernández-Poyatos et al. (2019) [28] found that *Berberis thunbergii* leaves under natural conditions accumulated more phenolic acids (e.g., chlorogenic up to 2-fold) than in vitro, due to abiotic elicitors enhancing C3H/C4H expression. Another relevant example is the study by Nguyen et al. (2024) [14], where metal stress in plants boosted phenylpropanoids via ROS-mediated pathway activation, mirroring our wild vs. in vitro differences.

These comparisons highlight the need for multi-elicitor strategies in in vitro systems to replicate wild pathway efficiency, with potential for scaling bioactive extracts in food metabolomics [20].

## 5. Conclusions

This study demonstrates that wild-growing *H. perforatum* and *P. oleracea* exhibit higher accumulations of phenolic compounds, vitamins, and selected secondary metabolites than in vitro cultures grown on MS medium supplemented with 25–50 µM MeJA. Because non-elicited in vitro controls were not included, our data primarily reflect differences between wild habitats and elicited in vitro cultivation and do not allow for the quantification of MeJA-specific effects. Future studies including non-elicited in vitro controls and different elicitor combinations will be needed to optimize the phenylpropanoid response. The phenylpropanoid pathway, central to these differences, appears to be more strongly activated by natural stresses in wild conditions, leading to higher levels of bioactives such as rutin, quercetin, caffeic acid, and chlorogenic acid, which are important for antioxidant potential in food applications.

Proximate, sugar, vitamin, and mineral composition further support this view: in vitro nutrient optimization enhances primary components (e.g., protein, minerals), whereas wild stresses favor secondary metabolism for resilience. These findings advance food metabolomics by positioning wild extracts as potent natural fortifiers for functional foods while indicating that in vitro systems can offer scalable, standardized alternatives when elicitation strategies are optimized. Future research should integrate multi-omics approaches and systematic elicitor screenings to refine in vitro elicitation regimes and better link pathway regulation to practical food bioactivity.

## Figures and Tables

**Table 1:** Physicochemical composition of herbal extracts, % (dry matter basis).

Parameter	Wild *H. perforatum*	*H. perforatum* In Vitro	Wild *P. oleracea*	*P. oleracea* In Vitro
Protein	45.46 ± 0.11 ^a^	49.20 ± 0.10 ^b^	34.35 ± 0.12 ^a^	38.10 ± 0.11 ^b^
Fat	16.75 ± 0.07 ^a^	18.10 ± 0.06 ^b^	8.29 ± 0.02 ^a^	8.90 ± 0.03 ^b^
Carbohydrates	31.67 ± 0.25 ^a^	32.65 ± 0.24 ^b^	31.61 ± 0.32 ^a^	34.80 ± 0.28 ^b^
Ash	6.12 ± 0.05 ^a^	7.05 ± 0.05 ^b^	25.75 ± 0.05 ^a^	28.20 ± 0.07 ^b^

Values are mean ± SD (*n* = 3). Different superscript letters within row indicate significant differences (ANOVA/Tukey’s HSD, *p* < 0.05).

**Table 2:** Total contents of polyphenols, flavonoids, and organic substances in herbal extracts, % (dry matter basis).

Parameter	Wild *H. perforatum*	*H. perforatum* In Vitro	Wild *P. oleracea*	*P. oleracea* In Vitro
TPC	2.70 ± 0.06 ^a^	3.58 ± 0.07 ^b^	1.25 ± 0.02 ^a^	1.78 ± 0.03 ^b^
TFC	2.35 ± 0.05 ^a^	3.12 ± 0.06 ^b^	0.98 ± 0.02 ^a^	1.35 ± 0.03 ^b^

Values are mean ± SD (*n* = 3). Different superscript letters within row indicate significant differences (ANOVA/Tukey’s HSD, *p* < 0.05).

**Table 3:** Characterization and quantification (mg/g for rutin/quercetin; mg/kg for acids) of phenolic compounds in extracts.

Plant	Rutin (mg/g)	Caffeic Acid (mg/kg)	Chlorogenic Acid (mg/kg)	Quercetin (mg/g)
Wild *H. perforatum*	22.224 ± 0.65 ^a^	31.23 ± 1.66 ^a^	10.79 ± 1.11 ^a^	1.773 ± 0.251 ^a^
*H. perforatum* in vitro	15.190 ± 0.311 ^b^	29.11 ± 1.48 ^b^	7.44 ± 0.81 ^b^	1.196 ± 0.166 ^b^
Wild *P. oleracea*	35.440 ± 4.120 ^a^	12.6 ± 0.2 ^a^	23.76 ± 1.89 ^a^	0.874 ± 0.157 ^a^
*P. oleracea* in vitro	20.445 ± 2.454 ^b^	<1.0 ^b^	13.79 ± 1.55 ^b^	0.444 ± 0.157 ^b^

Values are mean ± SD (n = 3). Different superscript letters within column for each species indicate significant differences (ANOVA/Tukey’s HSD, *p* < 0.05).

**Table 4:** Free carbohydrate content in herbal extracts, g/100 g.

Carbohydrate	Wild *H. perforatum*	*H. perforatum* In Vitro	Wild *P. oleracea*	*P. oleracea* In Vitro
Sucrose	0.45 ± 0.02 ^a^	0.52 ± 0.03 ^b^	nd	0.18 ± 0.02 ^b^
Glucose	1.60 ± 0.06 ^a^	1.98 ± 0.06 ^b^	2.28 ± 0.07 ^a^	2.75 ± 0.08 ^b^
Fructose	2.43 ± 0.07 ^a^	3.05 ± 0.08 ^b^	nd	nd

Values are mean ± SD (*n* = 3); nd = not detected. Different superscript letters within row indicate significant differences (ANOVA/Tukey’s HSD, *p* < 0.05).

**Table 5:** Vitamin content in herbal extracts, mg/100 g.

Vitamin	Wild *H. perforatum*	*H. perforatum* In Vitro	Wild *P. oleracea*	*P. oleracea* In Vitro
B1	3.781 ± 0.756 ^a^	4.920 ± 0.984 ^b^	2.150 ± 0.430 ^a^	2.780 ± 0.556 ^b^
B2	2.940 ± 1.234 ^a^	3.860 ± 1.617 ^b^	1.820 ± 0.764 ^a^	2.350 ± 0.987 ^b^
B3	11.703 ± 2.340 ^a^	14.850 ± 2.970 ^b^	7.450 ± 1.490 ^a^	9.620 ± 1.924 ^b^
B5	6.278 ± 1.256 ^a^	8.200 ± 1.640 ^b^	4.120 ± 0.824 ^a^	5.320 ± 1.064 ^b^
B6	1.135 ± 0.227 ^a^	1.490 ± 0.298 ^b^	0.850 ± 0.170 ^a^	1.100 ± 0.220 ^b^
B9	0.410 ± 0.082 ^a^	0.540 ± 0.108 ^b^	0.280 ± 0.056 ^a^	0.360 ± 0.072 ^b^
C	256.14 ± 87.08 ^a^	326.00 ± 111.0 ^b^	85.20 ± 34.08 ^a^	110.00 ± 44.00 ^b^

Values are mean ± SD (*n* = 3). Different superscript letters within row indicate significant differences (ANOVA/Tukey’s HSD, *p* < 0.05).

**Table 6:** Macro- and microelement contents in herbal extracts, mg/100 g.

Element	Wild *H. perforatum*	*H. perforatum* In Vitro	Wild *P. oleracea*	*P. oleracea* In Vitro
Fe	1.240 ± 0.004 ^a^	0.970 ± 0.003 ^b^	20.50 ± 0.05 ^a^	27.12 ± 0.06 ^b^
Zn	6.83 ± 0.31 ^a^	8.21 ± 0.33 ^b^	4.50 ± 0.10 ^a^	5.50 ± 0.12 ^b^
Mg	2.30 ± 0.02 ^a^	2.96 ± 0.03 ^b^	750.0 ± 1.5 ^a^	930.2 ± 0.8 ^b^
K	181.6 ± 0.8 ^a^	214.7 ± 0.9 ^b^	5500.0 ± 5.0 ^a^	6810.44 ± 2.45 ^b^
Ca	89.73 ± 1.07 ^a^	102.80 ± 1.14 ^b^	700.0 ± 2.0 ^a^	884.75 ± 1.50 ^b^
P	222.13 ± 0.09 ^a^	253.45 ± 0.10 ^b^	500.0 ± 1.0 ^a^	612.40 ± 1.20 ^b^

Values are mean ± SD (*n* = 3); Different superscript letters within row indicate significant differences (ANOVA/Tukey’s HSD, *p* < 0.05).

## Data Availability

The original contributions presented in this study are included in the article. Further inquiries can be directed to the corresponding author.
